# The Use of Fam‐Trastuzumab Deruxtecan‐nxki in Treating ERBB2 Amplified Small Cell Lung Cancer Transformed From Non‐Small Cell Lung Cancer: A Case Report

**DOI:** 10.1155/crom/8836862

**Published:** 2026-05-03

**Authors:** Alan Schumann, Charisse Brown-Moran, Chung-Ting J. Kou

**Affiliations:** ^1^ Department of Medical Oncology, Brooke Army Medical Center, San Antonio, Texas, USA, bamc.amedd.army.mil; ^2^ Department of Pathology, Brooke Army Medical Center, San Antonio, Texas, USA, bamc.amedd.army.mil

**Keywords:** case report, ERBB2, SCLC, transformed

## Abstract

Histological transformation from non‐small cell lung cancer (NSCLC) to small cell lung cancer (SCLC) is a recognized mechanism of resistance to epidermal growth factor receptor (EGFR) tyrosine kinase inhibitors (TKIs), leading to poor prognosis and significant therapeutic challenges. We present a case of a 66‐year‐old female with de novo metastatic NSCLC harboring an EGFR mutation, RET rearrangement, and ERBB2 amplification, who experienced transformation to SCLC while on osimertinib. Subsequently, she exhibited primary refractory disease to both first‐line platinum doublet with immunotherapy and second‐line lurbinectedin. Given her transformed disease and continued ERBB2 amplification on next‐generation sequencing (NGS), the patient was initiated on trastuzumab deruxtecan (T‐DXd) at a dosage of 5.4 mg/kg intravenously every 3 weeks. The patient had minimal side effects and obtained a partial response with a progression‐free survival (PFS) of 13.1 months, better than historically poor prognosis seen in transformed SCLC. This case underscores the potential role of human epidermal growth factor receptor 2 (HER‐2) directed therapies, such as T‐DXd, in transformed SCLC.

## 1. Introduction

The landscape of lung cancer treatment has evolved significantly with the advent of targeted therapies, particularly for NSCLC. However, the transformation of NSCLC to SCLC presents a unique clinical challenge, often associated with poor prognosis and limited treatment options [1]. In recent years, attention has been drawn to the role of T‐DXd, an antibody‐drug conjugate composed of humanized anti‐HER2 monoclonal antibody linked to a topoisomerase I inhibitor payload [2]. T‐DXd use is documented in NSCLC but not in SCLC or transformed disease.

## 2. Case Report

**Figure 1:**
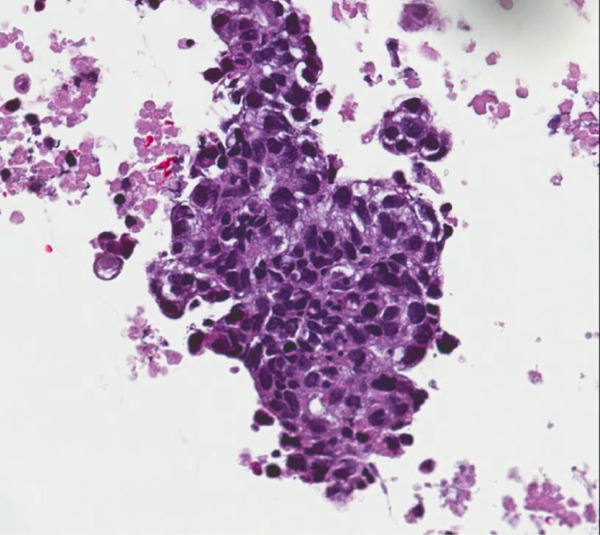
The cell block preparation of the supraclavicular lymph node demonstrates large, atypical cells with irregular nuclear membranes, H&E 400×.

**Figure 2:**
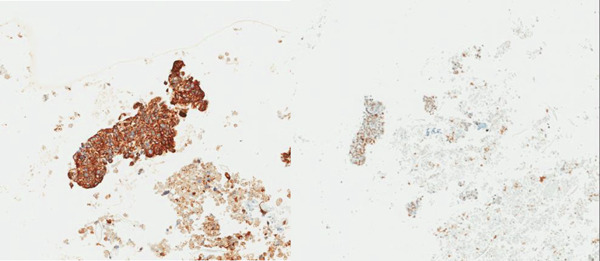
Immunohistochemical stains of the cell block preparation of the supraclavicular lymph node demonstrate positive staining for the cytokeratin Lu‐5, 200× (left) and for TTF‐1, 80× (right), supporting the diagnosis of adenocarcinoma, lung primary.

A 66‐year‐old woman presented with a 1.5‐month history of back pain. Initial imaging revealed a right upper lung mass, a nonobstructing hilar mass, and metastases to T8, L1 vertebrae, left fifth rib, and the liver without central nervous system involvement. Supraclavicular lymph node biopsy confirmed adenocarcinoma consistent with lung primary (Figure 1), showing positivity for Lu‐5, TTF‐1, CK7, and negativity for CK20, PAX‐8, and GATA‐3 (Figure 2). HER2 immunohistochemistry was not performed at initial diagnosis as the patient was diagnosed in December 2021 prior to the FDA accelerated approval of T‐DXd in NSCLC in August 2022. Next‐generation sequencing (NGS) identified an EGFR p.L858R missense variant gain of function mutation on exon 21, ERBB2 copy number gain, EPHA3‐RET rearrangement, rearrangement intron 11, tumor mutational burden 12.1 m/MB (90th percentile), MSI stable with PD‐L1 immunohistochemical staining of < 1%.

**Figure 3:**
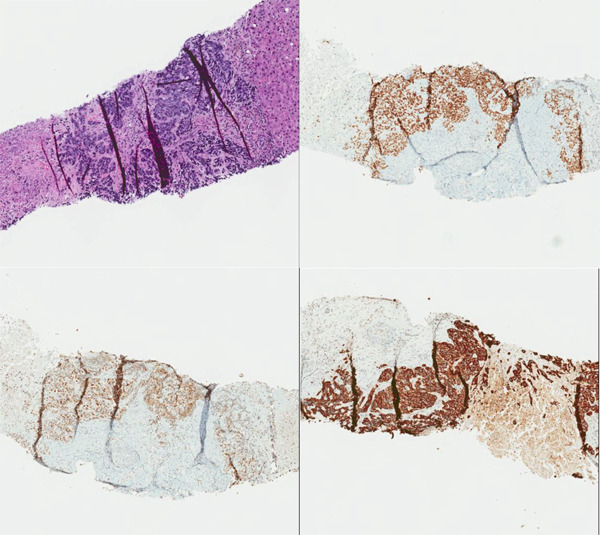
The core needle liver biopsy demonstrates infiltrative, pleomorphic epithelioid cells with surrounding necrosis and solid, trabecular architecture (top left, H&E, 80×). Special stains demonstrate positive staining for TTF‐1 (top right, 80×), INSM1 (bottom left, 80×), and Lu5 (bottom right, 80×).

The patient was initiated on osimertinib as first‐line therapy. Four months into therapy, the patient developed evolving lung masses with new liver and vertebral metastasis. Core needle liver biopsy showed hepatic parenchyma with infiltrative malignant epithelial cells with abundant necrosis and 40 mitotic figures per 10 high‐powered fields. Lesional cells were positive for TTF‐1, CK7, Lu‐5 (diffuse pattern), and INSM1 with a Ki‐67 proliferation index of 50%–75% (Figure 3). NGS demonstrated a new RB1 and TP53 mutation, while reestablishing prior findings: ERBB2 amplification, RET fusion, and EGFR mutation. Given histology and NGS findings with rapidly proliferating disease, the patient was diagnosed with transformation from NSCLC to SCLC.

Initially, the patient was treated with carboplatin, etoposide, and atezolizumab. However, the patient exhibited primary refractory disease on 3‐month follow‐up imaging, necessitating a switch to lurbinectedin. She again had primary refractory disease with progression on 3‐month follow‐up imaging.

**Figure 4:**
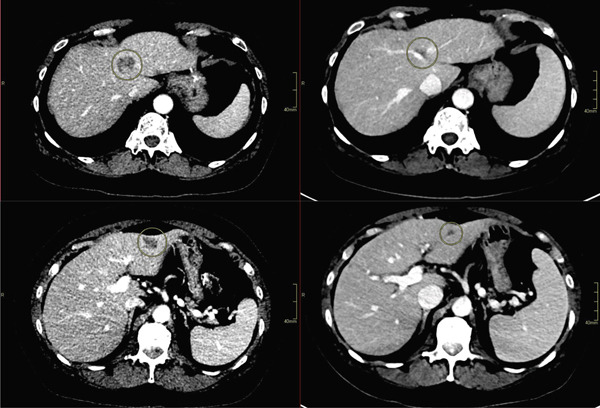
Axial contrast‐enhanced computed tomography (80/150‐kV peak) images demonstrate representative hepatic lesions in Segment IV (top row) and Segment III (bottom row) before (left, pretreatment) and after (right, posttreatment) therapy. Segment IV lesion size decreased from 2.1 × 2.0 × 2.0 cm pretreatment to 1.4 × 1.0 × 0.7 cm posttreatment. Segment III lesion size decreased from 2.0 × 1.2 × 1.3 cm pretreatment to 0.6 × 0.5 × 0.5 cm posttreatment.

We elected to trial T‐DXd 5.4 mg/kg IV every 21 days off‐label for ERBB2 amplification based on the results of DESTINY‐Lung 01 [2]. On 2 and 5‐month follow‐up imaging, the patient obtained a favorable partial response with decreased size of multiple pulmonary and hepatic lesions (Figure 4), mediastinal and hilar lymphadenopathy with stable osseous lesions with continued stability on follow‐up surveillance imaging. The patient experienced Grade 1 fatigue and Grade 2 dysgeusia with no dose adjustments needed.

The patient presented 13.1 months after initiating T‐DXd with new tension headaches, with subsequent MRI brain showing numerous enhancing intracranial lesions with superimposed leptomeningeal and pachymeningeal enhancement, with otherwise stable disease on systemic imaging.

In setting of her RET‐fusion, the patient was trialed on Selpercatinib with some data showing efficacy in leptomeningeal disease for NSCLC [3]. However, within 2 weeks of initiating therapy, the patient developed epigastric pain with radiographic concern for cholecystitis with Grade 3 elevated bilirubin with word‐finding, fatigue and difficulty with gait. The patient was ultimately transitioned to hospice.

## 3. Discussion

The transformation of NSCLC to SCLC, though relatively rare, is a well‐documented phenomenon. As seen in our case, SCLC histological transformation has been observed to occur 3%–14% in the context of acquired resistance to targeted therapies, such as EGFR inhibitors [1]. Median time to transformation is 16–19 months with overall survival (OS) after transformation being poor at 6–11 months [1]. Transformation from NSCLC to SCLC represents a significant clinical challenge due to the aggressive nature and distinct therapeutic requirements of SCLC compared with its non‐small cell counterpart.

The molecular mechanisms underlying the transformation are not fully understood, but several factors have been implicated. Genetic alterations, such as the loss of tumor suppressor genes, TP53 and RB1, are commonly observed and implicated in transformed SCLC [4, 5]. This transformation results in a change in cellular morphology and behavior, making the disease more aggressive and often more resistant to conventional NSCLC therapies.

The role of ERBB2 as a therapeutic target in NSCLC has been increasingly recognized with studies demonstrating the efficacy of HER2‐targeted therapies. The efficacy of T‐DXd in DESTINY‐Lung 01 was demonstrated in Cohort 2 in patients with HER2 mutations who received T‐DXd 6.4 mg/kg every 21 days with a median PFS of 8.2 months and a median OS of 17.8 months, with 46% of patients experiencing Grade 3 or higher adverse events. Responses according to Response Evaluation Criteria in Solid Tumors (RECIST) Version 1.1 were observed in both tumors with HER2 protein overexpression and tumors with *ERBB2* gene amplification [2]. ERBB2 amplification has been observed in 3%–5.6% of SCLC cases [4, 5]. However, the transformation of ERBB2‐amplified NSCLC to SCLC is less well‐characterized, and the therapeutic implications of maintaining HER2‐targeted therapy in this setting is unknown.

## 4. Conclusion

In this case, the use of T‐DXd in ERBB2 amplified transformed SCLC resulted in a significantly prolonged remission compared with historical data in transformed SCLC. There are currently no clinical trials specifically evaluating T‐DXd in SCLC, and to the best of our knowledge, no case report suggesting its potential benefit in this context. The biological rationale for its use in transformed SCLC hinges on the persistence of HER2 amplification as a therapeutic target even after histological transformation, which was the initial rationale for its implementation outside of conventional use.

Further research is vital to confirm benefits and standardize treatments. However, this case highlights the potential of targeted HER‐2 directed therapies, such as T‐DXd, to successfully treat transformed lung cancer.

## Author Contributions

Alan Schumann: conceptualization (lead), writing—original draft (lead), writing—review and editing (equal), and visualization (equal). Charisse Brown‐Moran: visualization (equal). Chung‐Ting J. Kou: writing—review and editing (equal).

## Funding

No funding was received for this manuscript.

## Disclosure

The views expressed in this abstract reflect the results of research conducted by the authors and do not necessarily reflect the official policy or position of Brooke Army Medical Center, the Defense Health Agency, Department of Defense, nor the US Government.

## Ethics Statement

Ethics approval is not applicable for this study.

## Consent

Written informed consent for publication of their clinical details and/or clinical images was obtained from the next of kin of the patient. A copy of the consent form is available for review by the editor in chief of this journal.

## Conflicts of Interest

The authors declare no conflicts of interest.

## Data Availability

Data sharing is not applicable to this article as no new data were created or analyzed in this study.
